# Neuromodulation of the subthalamic nucleus in Parkinson’s disease: the effect of fiber tract stimulation on tremor control

**DOI:** 10.1007/s00701-020-04495-3

**Published:** 2020-11-10

**Authors:** Arif Abdulbaki, Jörn Kaufmann, Imke Galazky, Lars Buentjen, Jürgen Voges

**Affiliations:** 1 Department Stereotactic Neurosurgery, University Hospital Magdeburg, Leipziger Str. 44, 39120 Magdeburg, Germany; 2 Department of Neurology, OvGU-Magdeburg, Magdeburg, Germany; 3 Center for Behavioral Brain Sciences (CBBS), Magdeburg, Germany

**Keywords:** Deep brain stimulation, Subthalamus, Dentato-rubro-thalamic tract, Fiber tracking, Parkinson’s disease, Tremor

## Abstract

**Background:**

Therapeutic effects of deep brain stimulation (DBS) of the subthalamic nucleus (STN) in Parkinson’s disease (PD) may in parts be attributed to the stimulation of white matter near the targeted structure. The dentato-rubro-thalamic (DRT) tract supposed to improve tremor control in patients with essential tremor could be one candidate structure. The aim of this study was to investigate the effect of stimulation proximity to the DRT on tremor control in PD patients treated with STN-DBS.

**Methods:**

For this retrospective analysis, we included 36 consecutive patients (median age 65.5 years) treated with STN-DBS for disabling motor symptoms including tremor. Stereotactic implantation of DBS electrodes into the motor area of the STN was performed using direct MRI-based targeting and intraoperative microelectrode recording. Tremor severity was assessed preoperatively and at regular intervals postoperatively (Unified Parkinson’s Disease Rating Scale III). The DRT was visualized in 60 hemispheres after probabilistic fiber tracking (3-T MRI). The position of active electrode contacts was verified on intraoperative stereotactic X-rays and postoperative CT images after co-registration with 3D treatment planning MRI/CT images. We determined the shortest distance of active contacts to the ipsilateral DRT tracts on perpendicular view slices and correlated this value with tremor change percentage.

**Results:**

Twelve patients had unilateral tremor only, and accordingly, 12 hemispheres were excluded from further imaging analysis. The remaining 60 hemispheres were associated with contralateral resting tremor. Active brain electrode contacts leading to resting tremor improvement (46 hemispheres) had a significantly shorter distance to the DRT (1.6 mm (0.9–2.1) [median (25th–75th percentiles)]) compared with contacts of non-responders (14 hemispheres, distance: 2.8 mm (2–4.6), *p* < 0.001).

**Conclusion:**

This retrospective analysis suggests that in STN-DBS, better tremor control in PD patients correlates with the distance of active electrode contacts to the DRT. Tractography may optimize both individually DBS targeting and postoperative adjustment of stimulation parameters.

## Introduction

The predominant aspects of the motor symptoms associated with Parkinson’s disease (PD) classify the patients into an akinetic-rigid, tremor-dominant, and balanced motor PD subtype. Patients with tremor-dominant PD present typically with rest tremor at a frequency of 4–6 Hz, which can be activated by mental stress and is temporarily suppressed during voluntary movements of the extremities.

Pathophysiological mechanisms supposed to be involved in tremor genesis are tremor of the extremity, tremor mediated through reflexes of the central nervous system (CNS), malfunction of feed forward loops within the CNS, and central oscillation. The latter is the result of rhythmic activity of a group of neurons within a nucleus or within neuronal networks, composed of loops consisting of neurons and their axons [17, 22]. Chronic high-frequency electrical stimulation (deep brain stimulation (DBS)) of the subthalamic nucleus (STN), the ventro-postero-lateral part of the globus pallidus internus (VPL-GPi), and the nucleus ventralis intermedius of the thalamus (V.im.) according to Hassler’s classification) reduces effectively Parkinsonian rest tremor. These anatomical structures are relay nuclei of the basal ganglia loop and/or the cerebello-thalamic loop. Even though the mode of action is not completely unraveled to date, one important therapeutic mechanism of DBS is the disruption and overriding of network-wide pathological signals leading to electrical stabilization of diseased neuronal networks. In this context, myelinated axons and hence white matter are the assumed target structures for DBS effects [31].

Velasco et al. proposed in 2001 the direct electrical stimulation of the prelemniscal radiation (RAPRL), a white matter tract in the subthalamic area, for DBS therapy of both tremor and rigidity in PD patients [40]. Others linked the motor improvement in PD patients following STN-DBS more generally to the white matter surrounding this small nucleus [23, 41] comprising the zona incerta (ZI) (anterodorsomedial to the STN), Forel’s field H, the lenticular fascicle (Forel’s H2 field anterodorsal to the STN), or the thalamic fascicle (H1 field dorsomedial to the STN). The RAPRL is located together with these anatomical structures, which, in essence, constitute the pallidothalamic bundle, in the posterior subthalamic area (PSA).

Another larger group of fibers inside the PSA belong to the dentato-rubro-thalamic (DRT) tract and represent ascending projections originating in the dentate (DN), emboliform, and globose nuclei of the cerebellum according to tracer studies in the brain of nonhuman primates [27]. The DRT fibers constitute the main fiber bundle of the superior cerebellar peduncle. Approximately 90% of DRT fibers decussate at the pontine region. The minority of these projections enters the contralateral RN, and the majority terminates in the thalamic ventralis oralis posterior nucleus (V.o.p.) and the V.im. These nuclei, in turn, project onto the primary motor cortex [24].

In the past, several groups proposed the PSA as probably optimum target area for DBS treatment of several tremor types [4]. Coenen et al. introduced specifically the DRT as candidate structure for effective antitremor neuromodulation [10–12, 14].

Two studies investigated systematically the antitremor effect of DRT neuromodulation on Parkinsonian tremor to date. Endpoints of these case series studies were tremor reduction either during 14 days postoperatively or immediately intraoperatively [14, 35]. At this early time point, however, tremor improvement resulting from micro-lesional effects cannot be excluded. Thus, we analyzed retrospectively in 36 consecutively operated PD patients the effects of STN-DBS on tremor at longer follow-up intervals and determined the spatial position of active electrode contacts relative to the DRT and hence a possible electrical co-stimulation of DRT fibers.

## Methods

### Patients

For this retrospective analysis, we included 36 consecutive patients (median age at surgery: 65.5 years (range 27–77 years)) treated with STN-DBS for PD. All patients suffered in the medication-off state from resting tremor. Other inclusion criteria were (i) treatment planning MRI examination with a 3-T scanner including diffusion-weighted imaging (DWI), (ii) at least one complete follow-up examination within the first year after surgery, and (iii) a score of ≥ 2 out of 4 in any resting tremor components of the Unified Parkinson’s Disease Rating Scale (UPDRS) III. Table 1 lists the main patients’ characteristics.

**Table 1:** Patient characteristics

Characteristic	Value
Median age in years (range) Median duration of disease in years (range) Median follow-up period in months (range)	65.5 (27–77) 7 (2–24) 3 (1–7)
Mean L-dopa test OFF score (± SD) Mean L-dopa test ON score (± SD)	31 (± 12) 16 (± 9)
Parkinson’s disease subtype (no. of patients)	
- Equivalent	20
- Tremor-dominant	12
- Akinetic-rigid	4

The clinical data and the tremor assessments were taken from the patients’ medical records. For the imaging analysis, we used the existing treatment planning MRI files stored in our database. All these patients gave their informed consent that their clinical and imaging data can be used in a strictly pseudonymous form for retrospective evaluations and publications.

### Surgical procedure

The standardized treatment planning MRI protocols for a 3-T scanner comprised T1-, T2-, and PD-weighted series and DWI. To obtain contrast-enhanced T1-weighted images, the patients received an intravenous contrast infusion.

STN targets were primarily defined using brain atlas coordinates (1.7 mm posterior, 4 mm ventral, and 10.5 mm lateral to mid-commissural point (MCP)). Then, we aligned axial T2-weighted images parallel to the AC-PC line (anterior-commissure-posterior commissure) and divided 4 mm below the intercommissural plane the hypointense STN signal into four quadrants. The definite target was placed at the intersection of these quadrants. If necessary, we adjusted the position in anterior-posterior direction in line with the rostral border of the nucleus ruber.

The surgery for stereotactic implantation of brain electrodes in local anesthesia (28 patients) or general anesthesia (8 patients) was basically performed as described previously [41]. Briefly, after mounting a modified Riechert-Mundinger stereotactic frame on the patient’s head, we performed an intraoperative contrast-enhanced stereotactic CT examination (intravenous infusion of 1.53 g imeprol/kg BW). Stereotactic CT scans were co-registered with images from preoperative MRI examinations using dedicated software (PraezisPlus, Heidelberg, Germany).

For intraoperative registration of local field potentials (LFPs), we used a microprobe positioning device with five parallel arranged tubes (MicroDrive, inomed, Emmendingen, Germany) and 3–5 micro−/macroelectrodes per patient. Microelectrode recordings (MER) started 5 mm above the planned target until reaching a position where the LFP pattern indicated the transition zone from the STN into surrounding white matter and/or substantia nigra (SN). In awake patients, targeting was completed by intraoperative neurological examination determining the minimum voltage required for maximal tremor suppression and/or the improvement of rigor and the threshold for stimulation side effects using trajectories with a MER signal typical for the STN motor area. In patients operated under general anesthesia, the propofol medication was stopped, prior to MER. Under continuous remifentanil medication, characteristic bursting activity of STN neurons started 10–15 min after that time point.

The definite brain electrodes were implanted under fluoroscopic guidance using the trajectory with the longest course in the STN motor area according to MER recordings and/or the largest therapeutic window (voltage for the induction of side effects, voltage for motor improvement). Fourteen patients received brain electrodes with four non-segmented contacts (ACTIVA model 3389, Medtronic Inc., MN, USA) and 22 patients quadrupolar brain electrodes with two segmented contacts ((Boston Scientific, MA, USA; Cartesia, directional lead, DB 2202 (*n* = 14)) or (St. Jude Medical Neuromodulation Division, Plano, TX, USA; Infinity™ DBS System, directional lead, 6170 and 6172 (*n* = 8))). In all brain electrodes, the distance between contacts was 0.5 mm. Electrodes were placed at the ventral border of the motor STN (reference: most distal contact in electrodes with non-segmented contacts and most distal segmented contacted (contact two out of four) in directional leads). We confirmed the electrode position intraoperatively on stereotactic X-ray images (anterior-posterior and lateral direction) using stationary biplanar X-ray tubes and additionally by macrostimulation and clinical examination.

Extension cables and impulse generators (ACTIVA-PC: 9 patients, ACTIVA-RC: 3 patients (Medtronic Inc., MN, USA), Vercise Gevia: 14 patients (Boston Scientific, MA, USA), Infinity 7: 8 patients (St. Jude Medical Neuromodulation Division, Plano, TX, USA)) were implanted at the same day under general anesthesia. All patients underwent postoperative non-stereotactic CT examinations. These images were co-registered with the stereotactic CT and preoperative MRI data.

### Tremor analysis

For the purpose of this study, we used in particular the resting tremor score of the UPDRS III motor score. The same expert movement disorders neurologist (I.G.) performed the examinations at baseline and postoperatively. We compared the preoperative OFF-medication state (OFF-MED) with the OFF-medication/ON-stimulation state (OFF-MED/ON-STIM) at follow-up visits. The global resting tremor score of the UPDRS III motor score rating all extremities plus lips and jaw can achieve a maximum value of 20 points. If resting tremor is registered separately for the right or left limbs, the maximum score is 8. For the actual tremor analysis, we defined a percentage value by dividing the patient’s individual score by 20 in the case of global resting tremor scores and by 8 in the case of limb resting tremor scores. Tremor response to DBS was defined as tremor percentage difference (subtraction of postoperative tremor percentage scores from corresponding preoperative values) (Table 2). Negative percentage values or 0% values were classified as “No Improvement.” Finally, the improvement tremor percentage was correlated with the distance to the DRT tract in the hemisphere contralateral to the affected upper limb. In 12 patients with only unilateral tremor, the hemispheres contralateral to the non-tremor body half were excluded from further analysis.

**Table 2:** Distances in mm between the active electrode contacts and the border of the DRT tract for limbs with resting tremor improvement (*N* = 46) and those without substantial improvement (*N* = 14) following DBS

	Distances							
	Mean	Min.	Max.	SD	Percentiles (median values in mm)			*p* value*
					25th	50th	75th	
Resting tremor improvement	1.8	0	6.3	1.4	0.9	1.6	2.1	< 0.001
No improvement	3.5	1.44	7.6	1.9	2.0	2.8	4.6	

*Mann-Whitney rank sum test

*min.* minimum, *max.* maximum, *SD* standard deviation

### MRI sequences, preprocessing, and fiber tracking

The imaging data were acquired on a Siemens MAGNETOM Verio 3-T MRI scanner (Siemens, Erlangen, Germany) using a 32-channel head coil and Syngo MR B19 software. The MR protocol includes a high-resolution, T1-weighted structural scan for anatomical reference using a 3D magnetization-prepared rapid acquisition gradient echo (MPRAGE) sequence (TE = 7.21 ms, TR = 2700 ms, TI = 1100 ms, flip angle = 7°, voxel size = 1 × 1 × 1 mm^3^, 176 axial slices, field of view = 256 × 192 mm^2^, scan time = 7 min:34 s) [32]. Moreover, a T2-weighted sequence (TE = 80 ms, TR = 6950 ms, bandwidth = 252 Hz/pixel, field of view = 256 × 192 mm^2^, 80 axial slices aligned with the anterior commissure-posterior commissure plane, slice thickness = 2 mm, scan time = 7 min:11 s) was acquired. DWI images were obtained using a twice refocused, single shot, echo planar imaging (EPI) pulse sequence [36] using the following parameters: TE/TR = 86/11900 ms, matrix size = 128 × 128, 80 contiguous axial slices, isotropic resolution = 2 × 2 × 2 mm^3^, receiver bandwidth = 1698 Hz/pixel, and an echo spacing = 0.69 ms. Diffusion-weighted volumes were acquired along 30 non-collinear diffusion directions with a *b* value *b* = 1000 s/mm^2^ and one volume without diffusion weighting (*b* = 0 s/mm^2^). We allowed for parallel acquisition of independently reconstructed images using generalized auto calibrating, partially parallel acquisitions or GRAPPA [21], with acceleration factor of 3 and 57 reference lines. The total acquisition time was 7 min:08 s. For correction of geometric distortions in EPI caused by magnetic field inhomogeneities, a B0 field map was acquired prior to the EPI sequence using a double-echo gradient recalled echo (GRE) sequence (TE ½ = 4.92 ms/7.38 ms, TR = 514 ms, flip angle = 60°, voxel size = 2.7 × 2.7 × 3.2 mm^3^, FoV = 256 × 256 mm^2^, 50 axial slices).

For preprocessing, we used the FMRIB software library (FSL, University of Oxford, https://fsl.fmrib.ox.ac.uk) version 5.0.9 [29]. To correct for eddy-current-induced distortions, the DWI images were registered to a corresponding non-diffusion-weighted volume based on a 12-dof affine transformation using *eddy_correct* with spline interpolation [20]. Geometric distortions induced by magnetic field inhomogeneities were corrected based on the GRE field map, and the diffusion data were registered to the corresponding structural scan. These steps (EPI distortion correction and EPI-to-MPRAGE registration) were performed simultaneously using *epi_reg*. Diffusion tensors were fitted with *dtifit* to obtain the eigenvalues and eigenvectors for each voxel.

**Fig. 1:**
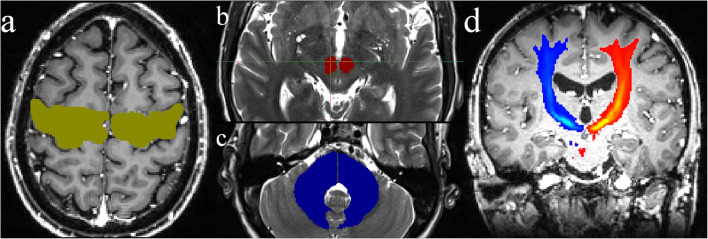
Regions of interest (ROIs) used for fiber tracking of the DRT. **a** Primary motor cortex. **b** Ipsilateral red nucleus. **c** Contralateral dentate nucleus. **d** DRT tract density map

The DRT reconstruction was carried out using a probabilistic approach [6] implemented in MATLAB (MathWorks, Natick, MA) in the native diffusion data space. Seed and filter regions were manually delineated based on structural T1- and T2-weighted images according to Kwon et al. and Yamada et al. [30, 42]. Conducting the stored spatial transformations, the region of interests (ROIs) were transformed in the native diffusion space. Briefly, DRT fibers started in the primary motor cortex as a seed region (precentral gyrus, 100,000 starts per voxel) and had to cross the following filter regions: ipsilateral red nucleus and contralateral dentate nucleus (Fig. 1). Hit maps were created by incrementation of each voxel (starting at zero) if it crossed a filtered path. The hit maps were transformed into the space of the T1 anatomy, and DICOM files were created using the Mathematica software package (Wolfram Research, Oxfordshire, UK).

To delineate the DRT, we used a method based on P-FT at which the likelihood of water molecules’ diffusion at multiple directions is determined [6] in comparison with only one direction per voxel as the case with deterministic fiber tracking (D-FT) [3, 16, 28]. Accordingly, the limitation of not being able to accurately delineate the white matter tracts in dense areas of crossing, kissing, or branching fibers was prevented. In accordance with groups that used P-FT [1, 9, 35], we were able to show the tract’s decussation instead of ipsilateral tracts resulted from D-FT [10–15, 19]. In fact, studies comparing P-FT and D-FT showed higher anatomical precision using P-FT not only with tracking the DRT but also other white matter fibers such as the corticospinal tract and fornix [8, 18, 38].

### Image analysis

**Fig. 2:**
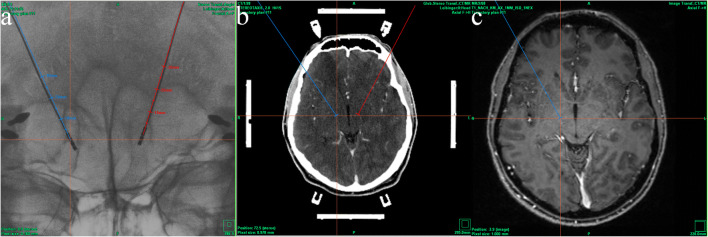
Co-registration of intraoperative stereotactic X-ray image (**a**) with stereotactic CT image (**b**) and preoperative MRI image (**c**)

Intraoperative stereotactic X-ray images (AP and lateral view) were co-registered with preoperative MRI images and intraoperative stereotactic CT images (Fig. 2). On the intraoperative stereotactic X-ray images, we registered for each active electrode contact X, Y, and Z coordinates, referring to the center of these contacts (monopolar cathodic stimulation). In the case of bipolar stimulation, we used the center of the distance between the contacts as reference. The obtained coordinates were transferred into the preoperative MRI images, which were co-registered with stereotactic CT images.

**Fig. 3:**
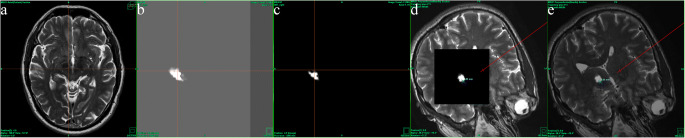
An illustration of our image analysis. **a** On axial T2-weighted MRI images 4 mm ventral to the intercommissural line, straight lines were inserted along the medial and posterior borders of the STN. **b** Tractographic MRI image displaying the DRT before contrast adjustment. **c** Tractographic MRI image displaying the DRT after contrast adjustment. **d** and **e** The shortest distance between the active contact and the DRT tract on images sectioned perpendicular to the long axis of the brain electrode was measured

Segmentation of the dentate nucleus was not very specific. As a result, the generated ROI included other cerebellar nuclei, contributing also fibers to the origin of the DRT. This wide segmentation resulted on DRT tracts which became visible on tractographic MRI images for each individual hemisphere with different contrast and consecutively different diameters. To standardize the contrast among study patients, we applied the following semiquantitative procedure: First, in each brain hemisphere, we defined on axial T2-weighted MRI images 4 mm ventral to the intercommissural line a straight line along the medial and posterior borders of the STN, respectively. Then, we adjusted the image contrast as long as the DRT tract signal did no more cross these lines. Finally, we measured the shortest distance between the active contact and the border of the DRT tract on images sectioned perpendicular to the long axis of the brain electrode (Fig. 3). Values were given in millimeters.

### Statistical analysis

Pairwise comparison between resting tremor percentage before and after DBS surgery was calculated using the Wilcoxon signed-rank test since the data did not pass normality tests. According to normality tests, independent *t* test or Mann-Whitney test was used to investigate difference of UPDRS III score between tremor responders group and tremor non-responders group. Mann-Whitney test was also used to determine significant difference of distances between the active electrode contacts and the DRT tract in both groups. Spearman’s correlation coefficient (Rs) was used to investigate correlation between resting tremor improvement percentage and distance to the DRT. The variances explained by the correlations (Rs^2^) were also calculated and reported. Significance was considered if the *p* value was ≤ 0.05. The statistical analysis was performed using SPSS 26 (statistical Package for the Social Sciences, IBM).

## Results

### General outcome

Implantation of DBS systems was performed without intraoperative or immediate postoperative complications. Three patients presented postoperatively with the following problems: suture granuloma in one case requiring wound revision and infection in two patients with subsequent removal of extension cables and impulse generator. The DBS systems were reimplanted 2 months later, which was in one case combined with repositioning of one brain electrode. The remaining 33 patients had no complications due to DBS surgery. The median postoperative follow-up time was 3 months (range, 1–7 months).

Impulse generator settings associated with the best individual improvement of motor symptoms were mean voltage 2.3 V (range: 1.1–4.0 V) for 14 patients with non-segmented electrodes, mean amplitude 3 mA (range: 1–5.5 mA) for the remaining 22 patients with segmented electrodes, mean pulse width 63.3 μs (range: 60–90 μs), and mean frequency 141.2 Hz (range: 130–210 Hz). We used monopolar cathodic stimulation in 63 electrodes, segmented monopolar cathodic stimulation in 5 electrodes and double monopolar cathodic stimulation in 3 electrodes, and segmented double monopolar cathodic stimulation in one electrode.

### Motor response—tremor

**Fig. 4:**
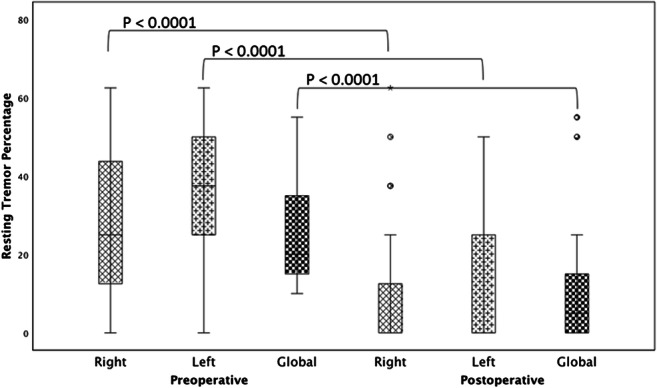
Improvement of the resting tremor UPDRS III scores under DBS (follow-up time: 1–7 month) compared with baseline. Displayed are percentage changes for right and left arm and the global percentage change. Whiskers represent range (minimum–maximum). Wilcoxon signed-rank test was used to determine significance

Compared with baseline, DBS reduced the resting tremor percentage significantly in the whole study population. The median global tremor percentage decreased from 20% (15–35) [25th–75th percentiles] preoperatively to 5% (0–15) postoperatively (*p* < 0.0001). Moreover, the median tremor percentage of right and left limbs decreased from 25% (12.5–46.9) [25th–75th percentiles] at baseline to 0% (0–12.5) under STIM-ON conditions (*p* < 0.0001) and from 37.5% (25–50) [25th–75th percentiles] to 0% (0–25) (*p* < 0.0001), respectively (Fig. 4). All differences were statistically significant.

### Motor response—UPDRS III scores stratified for patients with tremor improvement and patients with non-tremor improvement

**Fig. 5:**
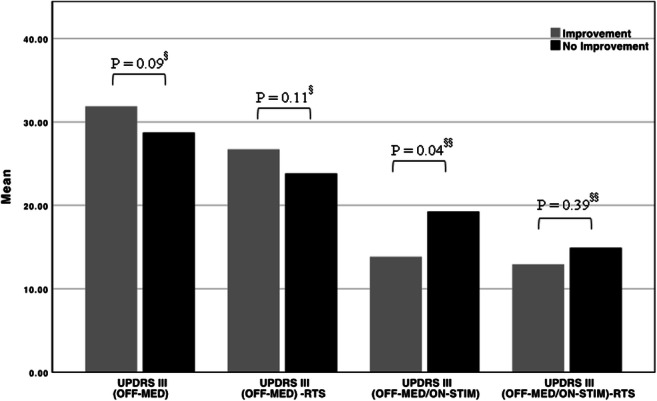
Bar graph displaying the mean UPDRS III scores of patients with tremor improvement under DBS (tremor responders, *N* = 25) and those with no tremor improvement (tremor non-responders, *N* = 11) in the preoperative OFF-medication state (OFF-MED) and postoperative OFF-medication/ON-stimulation state (OFF-MED/ON-STIM). Resting tremor score (RTS) components were subtracted from the total UPDRS III score at the different examinations time points. § Mann-Whitney test and §§ independent *t* test were performed to investigate statistical difference

At baseline, the UPDRS III motor scores in the OFF-medication state (OFF-MED) did not differ significantly between patients, with tremor improvement under DBS (tremor responders, *N* = 25) and those with no tremor improvement (tremor non-responders, *N* = 11) (tremor responder 31.88 ± 8.64 (mean ± SD) vs. tremor non-responder 28.73 ± 18.40 (mean ± SD), *p* = 0.09). When UPDRS III tremor scores were subtracted from the total UPDRS III score, the difference between the groups was also not statistically significant (*p* = 0.11). However, at follow-up visits, the UPDRS III motor score differed statistically significant between tremor responders (13.84 ± 6.49 (mean ± SD)) and tremor non-responders (19.27 ± 8.51 (mean ± SD), *p* = 0.04). After subtracting the resting tremor score from the follow-up total UPDRS III motor score (OFF-MED/ON-STIM), the group difference was again not statistically significant (tremor responder: 12.92 ± 6.06 (mean ± SD) vs. 14.91 ± 6.86 (mean ± SD) tremor non-responder, *p* = 0.39) (Fig. 5).

### Distance of active contacts the DRT and tremor response

Probabilistic fiber tracking of the DRT tract was performed in 60 hemispheres. Twelve hemispheres were excluded from imaging analysis because of absent tremor in the corresponding, contralateral body half at baseline. In 46 out of 60 hemispheres, DBS was associated with contralateral tremor improvement. In the remaining 14 hemispheres, electrical stimulation did not reduce contralateral tremor.

**Fig. 6:**
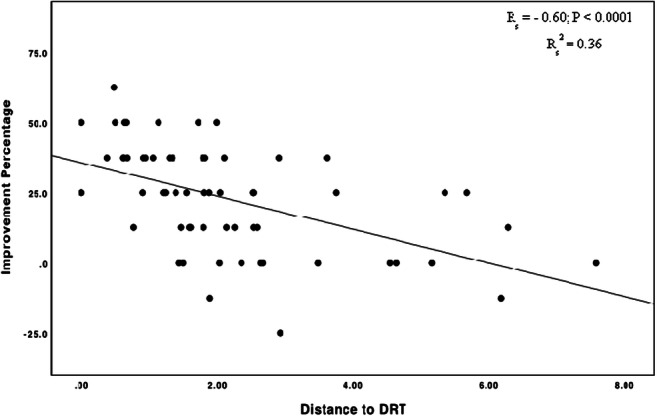
Relation between resting tremor improvement and distance of active electrode contacts to the DRT in mm. R_s,_ Spearman’s correlation coefficient; R_s_^2^, coefficient of determination

The distance between the active electrode contacts and the DRT tracts was shorter in tremor responders (1.6 mm (0.9–2.1) [median (25th–75th percentiles)]) compared with patients without tremor response (2.8 mm (2–4.6)) (Table 2). This difference was statistically significant (*p* < 0.001). Spearman’s correlation revealed a significant inverse correlation between resting tremor improvement percentage and distance to the DRT tract (R_S_ = − 0.6), *p* < 0.0001) (Fig. 6).

## Discussion

The main results of the actual study are (i) STN-DBS reduced resting tremor significantly in the whole study population. (ii) The measured distance was significantly shorter in brain hemispheres that corresponded to tremor improvement in the contralateral body half. (iii) Tremor improvement was significantly correlated to a shorter distance to the DRT tract. (iv) The antitremor effect seemed to be determined rather by the distance to white matter than by the position of active electrode contacts inside the motor area of the STN. When we subtracted the tremor subscore of the UPDRS III from the total UPDRS III score, there was no more a significant difference in the motor outcome between patients with tremor reduction after STN-DBS and those classified as tremor non-responders. We took this observation as an indirect argument that all patients underwent basically effective electrical stimulation in the STN motor part with the exception of tremor.

Table 3 summarizes the key findings of published studies addressing the role of electrical DRT stimulation and tremor control. In five of these case series, patients with ET were treated. With one exemption, the results confirmed the hypothesis that a shorter distance between active electrode contacts and the DRT may improve tremor suppression. Fenoy et al., who enrolled 20 ET patients in a prospective observational study, targeted the DRT directly. The volume of activated tissue of active contacts covered the DRT fibers partially or completely in all cases, and the tremor suppression under STIM-ON conditions was significant compared to baseline [19].

**Table 3:** A summary of the main published studies regarding the DRT involvement in tremor control

First author	Year	Number of patients	Target	Fiber tacking	ROIs	Key findings
Coenen	2011 [12]	Case report TD-PD	Thalamus (V.im.)	3-T MRI D-FT	DN Midbrain Precentral gyrus	First time to show the involvement of the DRT in tremor reduction through DBS
	2011 [10]	Case report dystonic head tremor (DT)	Thalamus (V.im.)			First time to use tractography in targeting DRT traverses the 3 targets (V.im., cZI, pSTN)
	2014 [11]	11 patients ET and PD and DT	Thalamus (V.im.)			In the case of excellent tremor control, effective electrode contacts projected onto the center of the DRT
	2016 [13]	2 patients PD	STN and DRT combined			The combined targeting approach appears to be safe and feasible
	2017 [15]	Case report ET	DRT in the STR			Revised surgery performed with DTI FT assistance Focused modulation of the DRT
	2020 [14]	36 pts. with various tremor types (8/36 PD pts.)	Subthalamic and thalamic portions of the DRT			DRT is potentially a common tremor-reducing structure
Schlaier	2015 [37]	5 patients ET	Thalamus (V.im.)	1.5-T MRI D-FT	DN S.C.P. RN	No sufficient evidence to use DRT as a new target for tremor
Anthofer	2017 [2]	6 patients ET	Thalamus (V.im.)			Shorter distance to the DRT in better responders
Calabrese	2015 [9]	12 patients ET	Thalamus (V.im.)	3-T MRI P-FT	S.C.P RN V.im.	Improvement correlated with the distance to the DRT not to the V.im.
O’Halloran	2016 [33]	2 patients PD	cZI	3-T MRI P-FT	Active electrode	2nd surgery for brain electrode repositioning using FT of DRT: tremor improved
Fenoy	2017 [19]	20 patients ET	DRT directly	3-T MRI D-FT	DN S.C.P Precentral gyrus	Direct DRT targeting suppresses tremor efficiently
Sweet	2014 [39]	9 patients PD	STN	3-T MRI D-FT	STN and RN Cerebellar hemisphere	Nonsignificant trend toward better control of PD motor symptoms including tremor with electrodes closer to the DRT
Prent	2019 [35]	20 patients PD	STN	3-T MRI CSD	DN S.C.P. RN	Activated contacts closer to the DRT showed increased tremor improvement.

*PD* Parkinson’s disease, *ET* essential tremor, *DT* dystonic tremor, *TD* tremor-dominant, *V.im.* ventral intermediate nucleus, *STR* subthalamic region, *cZi* caudal zona incerta, *D-FT and P-FT* deterministic and probabilistic fiber tracking, *CSD* constrained spherical deconvolution, *DN* dentate nucleus, *S.C.P.* superior cerebellar peduncle, *RN* red nucleus, *pts.* patients

Coenen et al. analyzed in a recently published retrospective study the relationship of symptom improvement after DBS and distance to the DRT in a heterogeneous group of patients with tremor of various origin (ET, PD, multiple sclerosis, dystonic head tremor, tardive dystonia). The authors used an efficacy measure, the tremor improvement per current ratio (TiCR = improvement on a four-point scale divided by the current applied in mA). The antitremor effect of DBS was determined intraoperatively at stimulation points with different distances to the target and hence to the DRT (251 points in total). The outcome measure TiCR deteriorated significantly if the distance to both the border and the center of the DRT increased [14].

Other studies listed in Table 3 aimed at the DBS treatment of Parkinsonian tremor only. In three of these studies, the patients underwent STN-DBS. The study from Sweet and coworkers aimed primarily at the tractographic visualization of fiber tracts connecting the basal ganglia with the cerebellum in 14 PD patients. In accordance with results from labeling studies performed in nonhuman primates, the group characterized in patients a descending subthalamo-ponto-cerebellar tract and the ascending dendato-thalamic tract. Nine out of 14 patients were treated with STN-DBS, and eight of nine presented with tremor. In these cases, the authors defined the proximity of active contacts and of the VTA of active electrode contacts to each of the fiber tracts. The relationship between motor outcome in general (tremor response was not specifically reported) and the distance from the active contact to the DRT was not significant. However, this group observed a nonsignificant trend toward better outcome when the VTA was closer to the fiber tract [39].

Prent et al. included in their retrospective study 35 patients treated with bilateral STN-DBS for PD. Twenty out of 35 patients were suffering from tremor. In these cases, the authors observed a statistically significant negative correlation between the proximity of active contacts to the DRT and tremor control 14 days after DBS surgery. The distance of electrode contacts associated with tremor improvement was located 1.13 mm closer to the center of the DRT compared with noneffective contacts [35]. This difference is approximately in the same range as in the actual study where effective electrode contacts were 1.7 mm closer to the border of the DRT compared with electrode contacts without antitremor effect. Moreover, the tremor improvement observed 14 days after implantation of brain electrodes by Prent et al. was not a result of microlesion effects but a DBS effect, because the actual study with longer follow-up intervals (range 1–7 months) came to the same conclusion.

As mentioned, the results of some studies on this subject were statistically not significant. Three possible reasons can probably explain this discrepancy. (i) The first possibility is the low sample size in many studies. The fact that electrical activation of the intended target area—the motor thalamus or the PSA—is supposed to have an antitremor effect leads a priori to a high number of tremor responders if electrical stimulation is performed. Consequently, differences of the strength of tremor reduction among the responding patients will be small which may complicate statistical comparison tests in small patient cohorts. Larger patient cohorts with a size comparable with the actual analysis may lead then to statistically significant results. (ii) The segmentation technique applied to tract the DRT can also potentially influence the measured distances. As noticed from Table 3, the definition of ROIs is heterogeneous among different study groups, limiting the direct comparison and assessment of the results published to date. (iii) Another possibility is that in contrast to the action tremor of ET patients, the proximity to the DRT will not exclusively determine the antitremor effect of DBS in resting tremor of PD patients. Helmich and others developed a dimmer-switch hypothesis of Parkinsonian tremor, which, in essence, has two driving components. One is located in the basal ganglia and represents the trigger of tremor. This part would explain tremor improvement following GPi-DBS. Referred to the subthalamic white matter tracts, candidate structures mediating antitremor effects of DBS could be the pallidothalamic projections (ansa lenticularis, lenticular fascicle). Activity inside the cerebello-thalamo-cortical circuit, on the contrary, is linked to tremor power and is supposed to act as tremor maintainer favoring the V.im. or the DRT as target [25, 26]. In addition, the role of the STN in Parkinsonian tremor is not completely explained by the dimmer-switch model. Anatomically, the STN has connections to the GPi, is directly connected to the motor cortex (hyperdirect path), and projects via the pons onto the cerebellum [7]. Thus, according to its particular position in the tremor circuitry, the STN could potentially be involved in triggering tremor, maintenance of the tremor rhythm, or in both [25].

Other white matter tracts inside the PSA with antitremor effects when electrically stimulated in PD patients are the caudal zona incerta (cZI) or the RAPRL located posterior and medial to the STN. Interestingly, three studies reported also a positive effect of DBS on other PD motor symptoms than tremor [5, 34, 40]. Blomstedt et al. randomized in their prospective study PD patients either to cZI-DBS or best medical treatment. The stimulation effect was clearly superior to medication, and the UPDRS III improvement of 41% (OFF-MED/ON-STIM) compared with baseline in the cZI-DBS group was on the order of other clinical studies randomizing patients to either STN-DBS or best medical treatment. However, in contrast to STN-DBS in patients with cZI-DBS, the dopaminergic medication could not be reduced significantly indicating probably a different mode of action [5].

Limitations of the actual study include the retrospective design and a tremor analysis performed unblinded and at different follow-up time points (range 1–7 month). Another critical point may be that our study cohort included patients with tremor-dominant PD and others with the balanced motor PD subtype. Patients with a tremor-dominant subtype have often a more benign disease course than patients without tremor. Even though there is, to our knowledge, no study addressing specifically the characteristics of tremor-dominant and balanced subtypes in PD, it cannot be excluded that the tremor of patients presenting with the balanced subtype may respond to STN-DBS differently compared with tremor-dominant patients.

In conclusion, this retrospective analysis is in line with the observation of others that in STN-DBS, a shorter distance of active electrode contracts to the DRT improves tremor control in PD patients. In three studies including the actual analysis, this correlation was statistically significant. As a consequence, the use of individualized tractography and identification of the distance between a planned trajectory and the DRT could potentially improve the outcome in PD patients treated with STN-DBS for tremor. In addition, further research combining advanced imaging and clinical data is necessary taking also candidate structures of the PSA other than the DRT into consideration.
